# Pre-amyloid oligomers of the proteotoxic RepA-WH1 prionoid assemble at the bacterial nucleoid

**DOI:** 10.1038/srep14669

**Published:** 2015-10-01

**Authors:** María Moreno-del Álamo, Susana Moreno-Díaz de la Espina, M. Elena Fernández-Tresguerres, Rafael Giraldo

**Affiliations:** 1 Department of Cellular and Molecular Biology, Centro de Investigaciones Biológicas – CSIC, Madrid E28040, Spain

## Abstract

Upon binding to short specific dsDNA sequences *in vitro*, the N-terminal WH1 domain of the plasmid DNA replication initiator RepA assembles as amyloid fibres. These are bundles of single or double twisted tubular filaments in which distorted RepA-WH1 monomers are the building blocks. When expressed in *Escherichia coli*, RepA-WH1 triggers the first synthetic amyloid proteinopathy in bacteria, recapitulating some of the features of mammalian prion diseases: it is vertically transmissible, albeit non-infectious, showing up in at least two phenotypically distinct and interconvertible strains. Here we report B3h7, a monoclonal antibody specific for oligomers of RepA-WH1, but which does not recognize the mature amyloid fibres. Unlike a control polyclonal antibody generated against the soluble protein, B3h7 interferes *in vitro* with DNA-promoted or amyloid-seeded assembly of RepA-WH1 fibres, thus the targeted oligomers are on-pathway amyloidogenic intermediates. Immuno-electron microscopy with B3h7 on thin sections of *E. coli* cells expressing RepA-WH1 consistently labels the bacterial nucleoid, but not the large cytoplasmic aggregates of the protein. This observation points to the nucleoid as the place where oligomeric amyloid precursors of RepA-WH1 are generated, and suggests that, once nucleated by DNA, further growth must continue in the cytoplasm due to entropic exclusion.

Factors modulating protein amyloidogenesis are the focus of intense research in the field of neurodegenerative diseases. Besides mutations and aberrant proteolytic processing of the proteins involved, ligands promoting amyloidogenesis, such as metal ions, glycosaminoglycans, phospholipids and nucleic acids have been recurrently reported as efficient co-factors for a number of proteins, most notably the mammalian prion PrP^1^,^2^,^3^,^4^. As first described by Jerson Silva and co-workers^5^,^6^,^7^ and extended by the group of Surachai Supattapone^8^,^9^,^10^,^11^, nucleic acids (DNA or RNA) can either act as efficient polyanionic macromolecular scaffolds or as allosteric effectors of PrP amyloidogenesis. However, much of the evidence on a role for nucleic acids in PrP amyloidogenesis relies on work performed *in vitro*, with little *in vivo* support so far.

RepA protein is a dual transcriptional repressor/DNA replication initiator encoded in plasmids from Gram-negative bacteria. In order to initiate DNA replication, RepA dimers, which are stable and soluble transcriptional repressors, must dissociate into metastable and aggregation-prone monomers. RepA dimers dissociate in response to their binding to specific DNA sequences from the replication origin, implying significant structural remodelling of the N-terminal domain (WH1). RepA-WH1 is thus converted from a dimerization domain into a DNA binding module, ancillary to the major determinant of sequence-specific DNA binding (the C-terminal domain, WH2)^12^,^13^,^14^,^15^.

Aiming to model *ab initio* protein amyloidogenesis within a synthetic minimal framework, rather than resorting to any of the known amyloidoses related to human disease, we engineered RepA-WH1 to rewire its conformational activation mechanism, generating a DNA-modulated amyloidogenic device. We used a RepA-WH1 variant carrying a mutation (A31V) conferring to the full length RepA enhanced capabilities in DNA replication^16^,^17^. We found that transient binding to short (11 bp) specific plasmid-derived dsDNA sequences *in vitro* modulated the assembly of the protein into amyloid fibres^18^. RepA-WH1 fibres are made of bundles of intertwined single or double filaments, with a hollow tubular core, in which distorted protein monomers stack along, and twist around, the axis of the filament^19^. A combination of *in silico* modelling and biophysical analyses led to the identification of a small molecule (tetra-sulphonated indigo, aka S4-indigo) that interfered with protein fibrillation, thus being a proof for the promotion by a nucleic acid ligand of protein amyloidogenesis *in vitro*^20^. Because DNA is not part of the fibres once these are assembled^18^, and the protein stretches directly involved in binding to nucleic acids and in amyloidogenesis are not coincident^20^, DNA acts as an allosteric effector of RepA-WH1 assembly.

Later on, we expressed in *E. coli* RepA-WH1(A31V), tagged with the fluorescent protein reporter mCherry, resulting in intracellular aggregation of the fusion protein^21^. These aggregates are distinct to the conventional inclusion bodies resulting from the expression of heterologous proteins in *E. coli*, qualifying RepA-WH1 as the first purely bacterial prionoid (i.e., a proteinopathic but non-infectious amyloid)^22^. This assertion is supported by: i) the acute cytotoxicity of RepA-WH1 aggregates; ii) their preferential staining with an amyloidotropic fluorophore; iii) their ‘vertical’ propagation from mother to daughter cells, driven by the high number of particles per cell; and iv) the existence of at least two alternative conformations/strains of RepA-WH1, whose interconversion is modulated by the Hsp70 chaperone DnaK^23^,^24^. As in the case of PrP^5^,^6^,^7^,^8^,^9^,^10^,^11^, the evidence for a role of dsDNA in RepA-WH1 amyloidogenesis *in vivo* is still indirect and feeble, including the enhancer effect on aggregation of the effector dsDNA sequence when cloned as multiple repeats in an expression vector^21^. However, this is not an absolute requirement, due to the presence in the *E. coli* genome of several sequences closely matching that of the DNA effector^21^. Development of new tools to survey nucleic acids-dependent RepA-WH1 amyloidogenesis *in vivo* is thus compulsory.

Although chemical probes such as Congo red, thioflavin-T and polythiophenes are widely used to screen for the amyloid state in protein assemblies^25^, antibodies specific for either oligomeric or fibrillar amyloid conformations are outstanding tools to screen, characterize and obtain insight into protein amyloidogenesis^26^,^27^,^28^,^29^. While some of these antibodies are able to recognize general features in the polymorphic amyloid cross-β structures, such as β-strand orientation or the distance between β-sheets, and are thus useful probes for the amyloids assembled by many different proteins^30^,^31^,^32^, others are specific for a particular protein. Overall, antibodies are most valuable as diagnostic and, potentially, therapeutic agents. Since amyloidogenesis of RepA-WH1 triggers a synthetic proteinopathy not naturally found in bacteria, and provides a platform to approach amyloid diseases in the simplest model system described so far, it is crucial to develop a specific probe to address issues such as the intracellular location where nucleation and assembly of these cytotoxic amyloid aggregates take place.

In this paper we describe the generation and molecular characterization of B3h7, a monoclonal antibody specifically recognizing a pre-amyloid oligomeric conformation of RepA-WH1. B3h7 revealed that, inside the *E. coli* cells, amyloid precursors are generated at the nucleoid, a finding compatible with the previously reported ability of dsDNA sequences to promote RepA-WH1 amyloidogenesis *in vitro*.

## Results

### B3h7: A monoclonal antibody specific for non-native RepA-WH1 conformations

**Figure 1:**
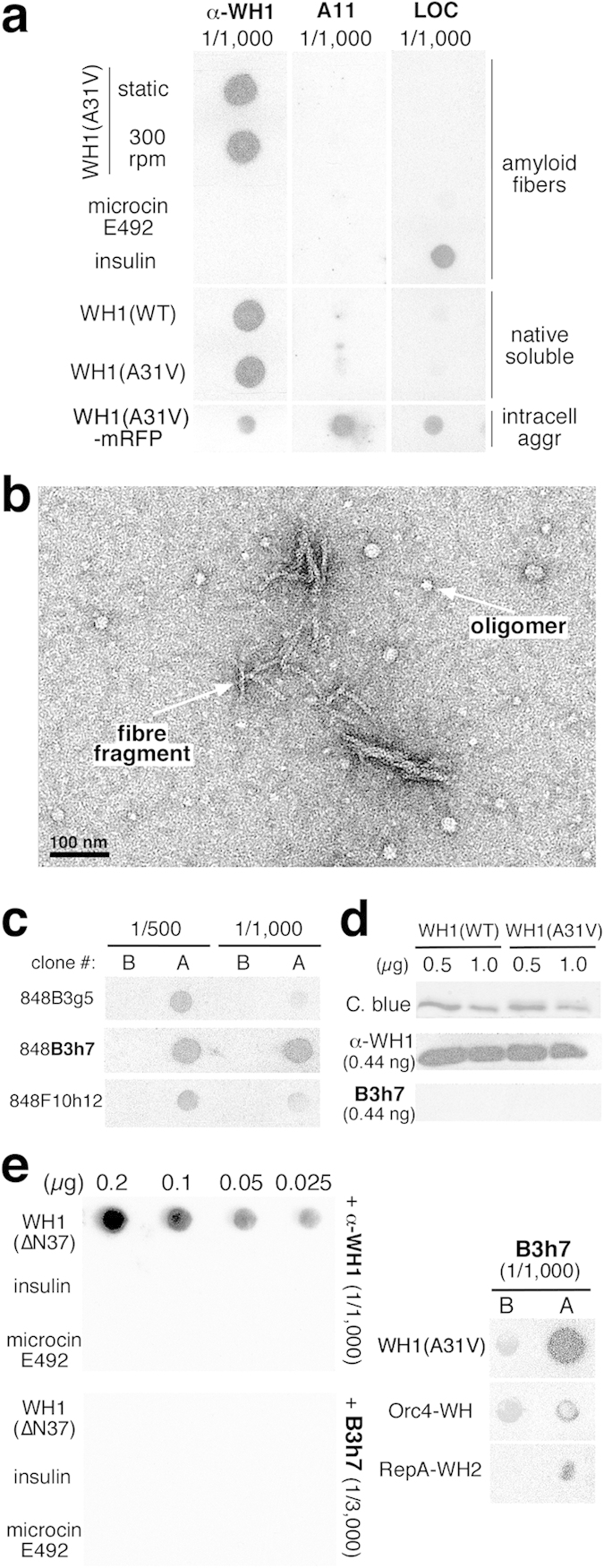
The mouse monoclonal antibody B3h7 targets assembled RepA-WH1. (**a**) The rabbit polyclonal antibodies α-WH1 (anti-RepA-WH1), A11^31^ (anti-amyloidogenic oligomers), or LOC^32^ (anti-amyloid fibres) were probed in dot-blot assays against RepA-WH1, insulin and microcin E492. Proteins blotted to the membrane (0.2 μg) were either native-soluble, assembled as amyloid fibres *in vitro*, or isolated *ex vivo* aggregates. The α-WH1 antibody, albeit having high affinity for RepA-WH1, did not discriminate between distinct conformations, whereas A11 and LOC did not recognize the RepA-WH1 amyloids assembled *in vitro*. Both antibodies reacted with the RepA-WH1-mRFP aggregates as extracted from bacteria, probably because these entrap other proteins exhibiting amyloid-like properties^58^. (**b**) EM micrograph of the immunogenic inoculum, obtained from sheared RepA-WH1(A31V) amyloid fibres and consisting of a mixture of small filaments and oligomers. (**c**) Dot-blot analysis of cell culture supernatants from three hybridoma clones exhibiting reactivity towards RepA-WH1(A31V) (oligomers plus sheared fibres mixture, 0.2 μg), immobilized either directly (A dots) or after boiling (B dots). The clone 848B3h7 (bold) was selected for further expansion on the basis of its higher affinity towards the assembled antigen. (**d**) Conformational specificity tested by Western blotting: B3h7 did not recognize the denatured RepA-WH1 bands, whereas α-WH1 did. (**e**) In dot blot assays, B3h7 was unable to recognize the mildly amyloidogenic RepA-WH1 deletion mutant ∆N37^12^,^22^,^23^, which was bound by the polyclonal antibody α-WH1 (*left*). Specificity of B3h7 was also tested against two structurally homologous WH domains, RepA-WH2^12^ and Orc4-WH^33^,^34^, which were weakly bound by the antibody (*right*).

We had previously generated α-WH1, a rabbit polyclonal antibody efficiently recognizing the native soluble and the aggregated conformations of RepA-WH1 in dot-blot assays (Fig. 1a), as well as its denatured state in Western-blots^23^. Commercially available anti-oligomer (A11)^31^ and anti-fibre (LOC)^32^ polyclonal antibodies were also tested against the native and amyloid conformations of RepA-WH1 but, in our hands, they showed limited affinity for the RepA-WH1 amyloid aggregates as extracted from bacteria, and virtually none for those assembled *in vitro* (Fig. 1a).

Therefore, we decided to develop a battery of monoclonal antibodies (MoAbs) in mice against the RepA-WH1 prionoid, with the hope of finding one of them that would target an amyloid-related conformation of the protein. We used as immunogen amyloid fibres of the hyper-amyloidogenic RepA-WH1 variant A31V^18^ that were mechanically sheared, by repeated pipetting through a narrow borehole micro-tip, to generate a mixture of oligomeric particles and filament fragments, as checked by means of EM (Fig. 1b). After serial mice immunization and several rounds of hybridoma subcloning, the antibody secreted by one of the isolated clones (B3h7) exhibited high affinity in dot-blot assays (Fig. 1c) for a mixture of assembled RepA-WH1(A31V) amyloids, but not when these were denatured before binding to the membrane, or in Western-blot assays after SDS-PAGE (Fig. 1d). Regarding B3h7 specificity, this antibody recognized neither aggregates of ∆N37, a deletion mutant of RepA-WH1^12^ that had been found to be mildly amyloidogenic and cytotoxic when compared with the RepA-WH1(A31V) variant^22^,^23^, nor amyloid fibres of insulin or microcin E492 (Fig. 1e, *left*). As further controls for specificity (Fig. 1e, *right*), B3h7 did not bind to the C-terminal domain of RepA (WH2)^12^, or to the WH domain of the yeast DNA replication protein Orc4p^33^,^34^, which are structurally related to RepA-WH1 but have reduced sequence identity and have not been reported to assemble as amyloids.

**Figure 2:**
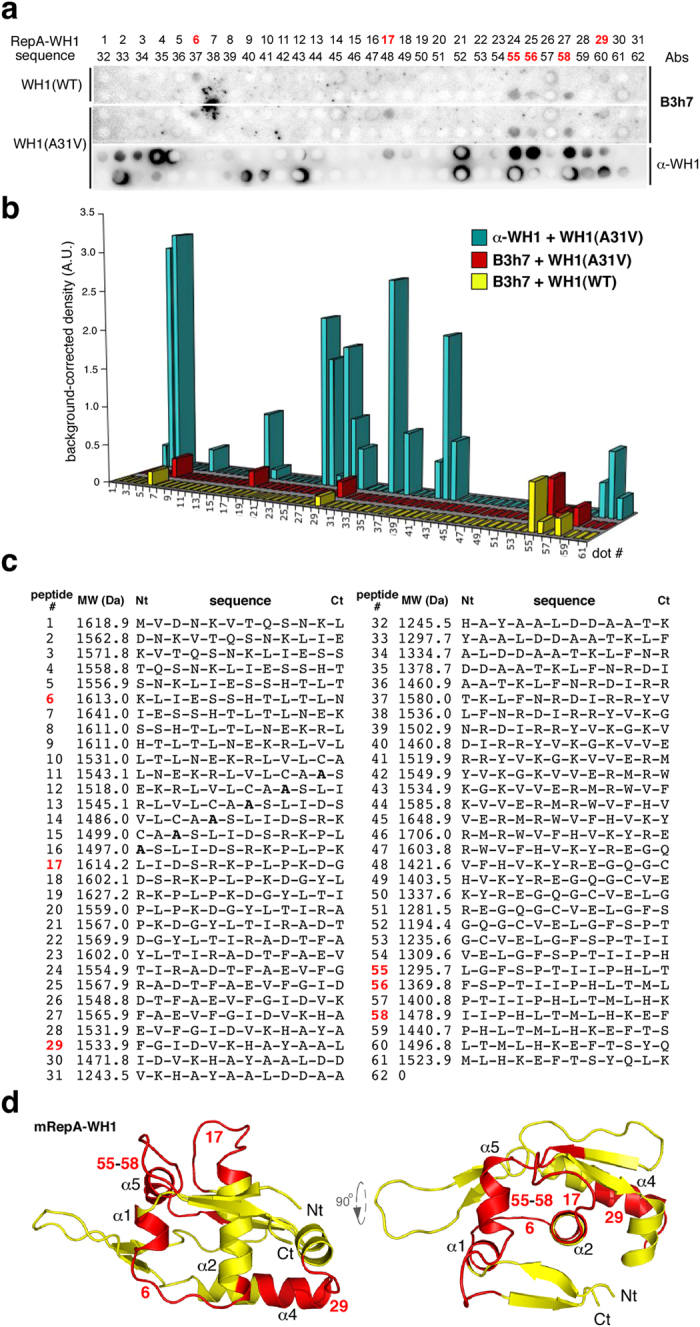
Mapping the epitope(s) for the B3h7 antibody in RepA-WH1. (**a**) Incubation of B3h7 and α-WH1 antibodies with a peptide array displaying the entire sequence of RepA-WH1 as partially overlapping 12 amino-acid residues. (**b**) Blots were quantitated and, upon normalization to the amount of peptide in each spot, the results were displayed as histograms. The intensities of the signal of B3h7 on the peptide spots were rather weak, as expected for a conformational antibody recognizing an epitope formed by parts from regions distant in the sequence of the protein, but close in its three-dimensional structure (see below), rather than linear epitopes as for α-WH1. (**c**) The sequences of the RepA-WH1 peptides in a, indicating their position number in the array. In bold, the Ala to Val mutation^16^,^17^ in the A31V version of the array. The six peptides recognized by B3h7 are displayed in red. (**d**) Two orthogonal views (rendered with PyMOL; http://www.pymol.org) of a model of the RepA-WH1 monomer, which builds the amyloid filaments by undergoing an increase in β-sheet^19^. Highlighted in red, the peptide stretches bound by B3h7: these cluster in two topologically defined regions in RepA-WH1, expanding α1 and α5 (peptides #6 and #55-58) and α4 (peptide #29), respectively. Peptide #17, corresponding to the loop with the highest B-factor (conformational flexibility) in the crystal structure of RepA-WH1^13^, has a central role in amyloidogenesis^18^. The two clusters are expected to assemble close together into a single conformational epitope upon the structural transformation linked to RepA-WH1 amyloidosis^18^,^19^.

Incubation of the B3h7 antibody with peptide arrays displaying the whole RepA-WH1 sequence on a membrane, either in its WT or A31V variants (Fig. 2a,b), revealed that the MoAb recognized an epitope made of up to four stretches spread across the RepA-WH1 sequence (Fig. 2c). Three of such peptides (# 6, 17, 55-58) were located close in the three-dimensional structure of the protein monomer, which is the molecular species that builds RepA-WH1 amyloid fibres^19^. However, the fourth sequence (# 29) was further apart (Fig. 2d), implying that the MoAb should recognize a distorted kind of RepA-WH1 monomer in which all four peptide stretches are clustered together; i.e., B3h7 indeed is a conformation-specific antibody. The requirement of a tetra-partite conformational epitope for high affinity B3h7 binding explains why the intensities of the hybridization signals for B3h7 on each of its constituent peptides were substantially weaker than those for the multiple linear epitopes recognized by α-WH1 (Fig. 2b). The peptide segments bound by B3h7 are coincident with regions undergoing structural rearrangements coupled to both the functional activation of RepA^13^ and RepA-WH1 amyloidogenesis^18^.

### B3h7 binds to amyloidogenic oligomers of RepA-WH1 inhibiting fibre assembly

**Figure 3:**
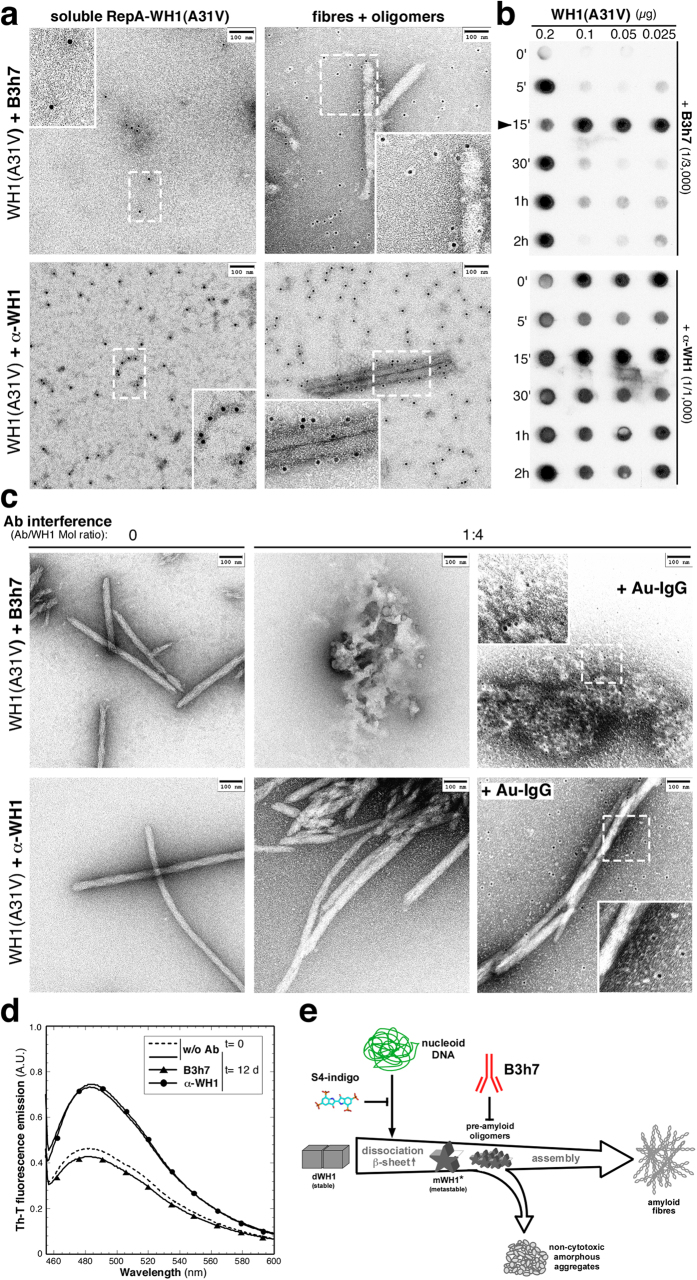
B3h7 is specific for pre-amyloid RepA-WH1 oligomers. (**a**) B3h7 (*top row*) and α-WH1 antibodies (*bottom row*) were incubated with native soluble (*left column*) or pre-assembled (*right column*) RepA-WH1(A31V), and then were probed with secondary gold-conjugated antibodies (electron-dense dots). Boxed sectors were magnified two-fold to highlight details. B3h7 barely binds to the soluble RepA-WH1, whereas α-WH1 recognizes it efficiently (*left*). On the assembled protein (*right*), both antibodies bind to oligomeric RepA-WH1 species (the unstained rim around each Au particle), but only α-WH1 recognizes the protein on the fibres. (**b**) Probing RepA-WH1 assembly kinetics. Samples were incubated with effector dsDNA and aliquots were drawn at the indicated intervals and dot-blotted before incubation with the antibodies. Arrowhead points to the sample (15 min) where the oligomeric precursors recognized by B3h7 peak. (**c**) B3h7 competes with the assembly of RepA-WH1(A31V) into amyloid fibres *in vitro*. Incubation with a sub-stoichiometric molar amount (1:4) of B3h7 (but not of α-WH1) for 12 days interfered with RepA-WH1(A31V) amyloidogenesis by causing accumulation of oligomers (Au-IgG labelled). B3h7 binding drove RepA-WH1 oligomers towards off-pathway amorphous aggregation but α-WH1, once titrated out by the excess of RepA-WH1, had no major effect on fibre assembly. (**d**) Th-T fluorescence revealed a substantial loss in RepA-WH1 amyloidogenicity linked to incubation with B3h7, as expected from the promotion of amorphous aggregation (see c), whereas α-WH1 had no significant effect. (**e**) A model for DNA (nucleoid; in green)-promoted amyloidogenesis of RepA-WH1. Starting from stable and soluble protein dimers, amyloidogenesis goes through dissociation into metastable monomers (WH1*), which assemble into pre-amyloid oligomers on pathway towards mature amyloid fibres. The specific inhibitors of RepA-WH1 amyloidogenesis S4-indigo^20^ and B3h7 MoAb (red) act preventing either the initial DNA-dependent monomerization or further assembly of the pre-amyloid oligomeric intermediates, respectively.

The B3h7 and the α-WH1 antibodies were purified and their ability to recognize RepA-WH1 amyloids assembled *in vitro* was then assayed by immuno-electron microscopy (iEM), labelling with gold (Au)-conjugated secondary antibodies (Fig. 3a). B3h7 did not bind to soluble RepA-WH1 dimers (Fig. 3a, *top row, left*), but to RepA-WH1 amyloid oligomers, either on-pathway intermediates or detached from the fibres, rather than to the fibres themselves (Fig. 3a, *top row, right*). B3h7 was also used in dot-blot assays to probe, under conditions of shaking-promoted fast kinetics, RepA-WH1 assembly along a time course (Fig. 3b, *top panel*): the label of the B3h7 antibody peaked at 15 min of incubation, to decrease thereafter when fibres were predominant. These results are consistent with the findings reported above (Fig. 2) on an epitope distribution for B3h7 compatible with distorted RepA-WH1 monomers, provided these were assembled as oligomers but not as mature fibres, rather than with native dimers. However, α-WH1 recognized RepA-WH1 whatever its assembly state (Fig. 3b, *bottom panel*), either as native dimers (Fig. 3a, *bottom row*, *left*), oligomers or fibres (Fig. 3a, *bottom row*, *right*), as expected for an antibody binding to discrete, linear epitopes (Fig. 2b).

Interestingly, co-incubation of B3h7 and RepA-WH1 during amyloidogenesis revealed that sub-stoichiometric amounts of the conformation-specific antibody inhibited the formation of amyloid fibres, according to electron microscopy observations in dsDNA-seeded reactions^18^ (Fig. 3c). Furthermore, binding of B3h7 drove the assembly of RepA-WH1 as amorphous aggregates (Fig. 3c, *top row*). However, at the same sub-stoichiometric ratios, α-WH1 did not interfere with fibre assembly and, once titrated-out by RepA-WH1, the fibres appeared unlabelled most likely due to the exhaustion of the pool of free antibody (Fig. 3c, *bottom row*).

In samples seeded with purified intracellular RepA-WH1 aggregates^21^, B3h7 efficiently reduced amyloid-specific labelling with the fluorophore thioflavin-T (Th-T) (Fig. 3d). The intensity of fluorescence emission when B3h7 had been included in the assembly reaction decreased by around 50% compared with samples in which fibre assembly was not challenged with the conformational antibody; i.e., Th-T emission decreased to nearly the same intensities observed for samples analysed immediately after setting-up the assays. However, if α-WH1 was the antibody used in the assay, fluorescence emission remained virtually unaltered as for mature fibres.

These results are compatible with a scenario in which the assembly of RepA-WH1 into amyloid fibres was competed by binding of the conformational antibody B3h7 to oligomeric particles and imply that such oligomers are obligate on-pathway intermediates^35^ in RepA-WH1 amyloidogenesis.

### Amyloid precursors of the RepA-WH1 prionoid are generated inside the bacterial nucleoid

**Figure 4:**
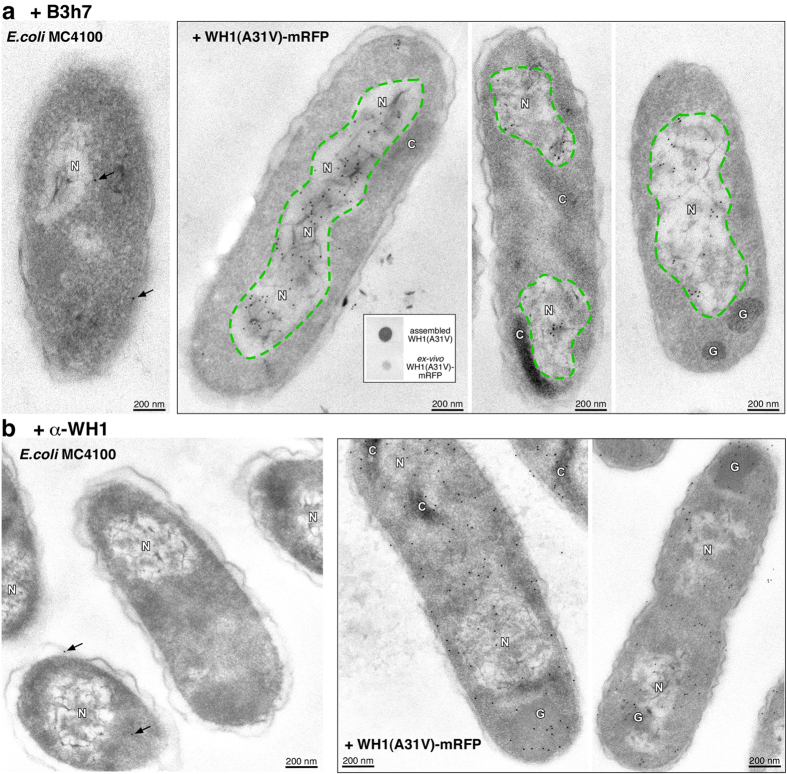
Oligomeric precursors of the RepA-WH1 prionoid are found in the nucleoid of *E. coli* cells. In both iEM panels, the left hand plate shows a control with cells not expressing RepA-WH1(A31V)-mRFP, but yet probed with the antibodies (arrows point to background Au-IgG). (**a**) According to the gold label (dots), the B3h7 antibody located the pre-amyloid RepA-WH1 oligomers as discrete inclusions inside the low contrasted nucleoid area (labelled as N; outlined green sectors). However, no labelling was found on the large, mature electron-dense RepA-WH1 aggregates, either globular (G) or comet-shaped (C)^21^,^23^. *Inset box*: dot-blot assay of B3h7 (0.4 ng) with 0.2 μg of *in vitro*-assembled RepA-WH1 fibres (*top*) and purified intracellular RepA-WH1-mRFP aggregates (*bottom*), which exhibited reduced labelling due to their low content of oligomeric species^21^,^23^. (**b**) α-WH1 labelling was found wherever RepA-WH1 was located, regardless of its conformation: on the large G/C aggregates and, with lower particle densities, also at the nucleoid and in the cytoplasm.

The B3h7 monoclonal antibody was then tested for the detection of possible amyloidogenic oligomers assembled *in vivo* by the RepA-WH1 prionoid. iEM was again the technique of choice, rather than immunofluorescence labelling, due to its much higher spatial resolution. On thin sections through *E. coli* cells that expressed the prionoid (Fig. 4a), Au-labelling was evident, thus locating the oligomer-specific B3h7 antibody, on small, mildly electron-dense subsectors inside the nucleoid area. B3h7 was not found on the large, mature RepA-WH1 amyloid aggregates, either in their characteristic globular (G) or comet-shaped (C) phenotypic variants/strains^23^, which were nucleoid-excluded. This is compatible with the observation that purified whole RepA-WH1-mRFP aggregates were poorly recognized by B3h7 in dot-blot assays (Fig. 4a*, inlay box*), since quantitatively the oligomers are minor species in the cells. In contrast, labelling with the polyclonal α-WH1 antibody (Fig. 4b) displayed the Au particles on both the large G and C-aggregate variants and the nucleoid, as expected from the ability of this antibody to bind to any RepA-WH1 molecule, regardless of its conformation. In *E. coli* cells not carrying RepA-WH1, because they lacked the expression vector (Fig. 4a,b; *left hand panels*), there was no significant labelling by either of the two antibodies, thus validating the specificity and sensitivity of the assay.

We have shown here that the monoclonal antibody B3h7, by targeting on-pathway oligomers, is a powerful tool for probing, and interfering with, amyloidogenesis of the synthetic RepA-WH1 prionoid *in vitro*. Beyond previous *in vitro* assays with purified RepA-WH1 and nucleic acids^18^,^20^, B3h7 has also provided evidence for the nucleoid as the physical place where DNA-promoted RepA-WH1 amyloidogenesis takes place inside bacteria, in itself a far-reaching observation for the physiological relevance of ligand binding in protein amyloidoses.

## Discussion

The results reported here provide evidence on the nucleoid, the complex subcellular structure that organizes the bacterial chromosome^36^, as the place where oligomeric particles of the synthetic prionoid RepA-WH1 are generated in *E. coli* (Fig. 4a). This observation is in agreement with the fact that the *E. coli* genome contains multiple copies of the DNA sequence that was previously found to be maximally efficient in promoting the amyloidogenesis of RepA-WH1^18^,^21^. It has been recently described that propagation of the RepA-WH1 prionoid depends on the DnaK (Hsp70) chaperone, which contributes to the generation of oligomeric aggregates (propagons)^23^. In fact, iEM with anti-DnaK antibodies located a subpopulation of molecules of the chaperone forming small clusters at the nucleoid^23^. It is thus likely that both cofactors, DNA and DnaK, work together on RepA-WH1 amyloidogenesis at the bacterial nucleoid. Since B3h7 is a probe specific for RepA-WH1 oligomers placed on the pathway towards mature amyloid fibres (Fig. 3e), it is likely that small amyloid precursors are generated inside the bacterial nucleoid, to become then entropically excluded towards peripheral cytoplasmatic regions upon further growth in size of the aggregates^37^,^38^. In terms of the average size reached by the RepA-WH1 particles before being excluded from the nucleoid, electron-dense protein aggregates of up to 100 nm were still retained within the boundaries of the nucleoid^23^. Once RepA-WH1 is assembled as amyloid fibres, B3h7 does not recognize the protein any longer (Fig. 3a,b), implying that some of the four parts of the conformational epitope for this antibody (Fig. 2), exposed in the oligomers, become otherwise arranged or inaccessible upon further assembly as fibres. Because the fourth peptide stretch contributing to build in RepA-WH1 the conformational epitope for B3h7 (#29, Fig. 2) is part of α4, the main DNA binding determinant in the protein^13^,^20^, assembly of the amyloid fibres might compete with DNA recognition, thus contributing to detach RepA-WH1 from the nucleoid.

Nucleic acid-promoted amyloidogenesis has been extensively studied *in vitro* for the mammalian prion protein, PrP^1^,^3^,^4^. In this case, both DNA and, specially, RNA have been implicated as amyloidogenic ligands. RNA is a potential cytoplasmic partner encountered by proteins that are internalised through endocytosis, such as Alzheimer’s Aβ peptides and Tau, Parkinson’s α-synuclein or PrP. Proteins involved in RNA processing, including FUS and TDP-43, have a nuclear localization and are linked to neurodegenerative diseases such as amyotrophic lateral sclerosis^39^. On the other hand, DNA would have its chance in proteins carrying poly-Gln expansions, such as Huntington’s Htt or the ataxins, whose aggregates usually have a nuclear localization^40^. The issues of mapping the intracellular location of distinct protein conformers along the pathway(s) of amyloid assembly, and the determination of their neurotoxic or benign nature, are thus hot topics usually addressed by using conformation-specific antibodies^41^,^42^,^43^,^44^. In a similar way, the monoclonal antibody B3h7, due to its specificity for pre-amyloid oligomers, has been a key probe to reveal that the bacterial nucleoid is where RepA-WH1 amyloidogenesis initially takes place.

Therapies for human amyloidosis based on circulating antibodies are actively sought in translational research^28^,^29^, with some notable achievements in animal models, such as the clearance of amyloid deposits^45^ or the mobilization of proteinopathic oligomers towards the generation of non-cytotoxic amorphous aggregates^46^. We reported earlier S4-indigo as a small molecule inhibitor of DNA-promoted amyloidogenesis of RepA-WH1 *in vitro*^20^. Although B3h7 shares with S4-indigo their ability to interfere with RepA-WH1 amyloidogenesis, albeit through different mechanisms, the former has the advantage of the feasibility of antibodies to be engineered by means of rational design and/or through combinatorial approaches. In particular, single chain antibodies from mammals (scFVs)^47^ or single domain nanobodies from camelids (VHHs)^48^ have been modified to improve their solubility, stability and selectivity. It is noteworthy that grafting amyloid stretches in the antigen recognition loops (CDRs) of scFVs has been recently described as a promising approach towards more efficient immunotherapies for human amyloidosis^49^,^50^. Furthermore, both scFVs and VHHs have been successfully expressed in *E. coli*, thus enabling their usage as intrabodies to detect specific intracellular proteins in bacteria and to interfere with their function and interactions^51^,^52^,^53^. Following this track, antibodies might play a role as devices in the development of synthetic circuits aiming to modulate intracellular bacterial amyloidosis, a relatively unexplored venue compared to what it is known about functional extracellular amyloids in bacteria^54^.

## Methods

### Assembly of RepA-WH1 amyloid oligomers and fibres

RepA-WH1(A31V) fibres were assembled *in vitro*, as described^18^, using the *opsp* 11 bp DNA sequence (5′ CATTGACTTGT/5′ ACAAGTCAATG) as amyloidogenic ligand (12.5 μM RepA-WH1 dimers and *opsp*), in 100 μl of 0.1 M Na_2_SO_4_, 25 mM Hepes·NaOH pH 8, 7% PEG4000 and 3% MPD, by standing still at 5 °C for 1 month. Alternatively, when indicated, agitation (600 rpm; Eppendorf Thermomixer) was performed during incubation, but reducing the concentration of dsDNA ten-fold, to speed-up the kinetics of amyloidosis from 2–4 weeks to 2 h, yielding fibres with the standard morphology^18^,^19^. For the experiments testing the interference of antibodies with RepA-WH1(A31V) amyloidogenesis, antibody aliquots were included at the indicated concentrations while assembling the samples. When the assembly of RepA-WH1 was monitored with Th-T (see below), amyloidogenesis was triggered with 10 ng of RepA-WH1(A31V)-mCherry seeds^21^, thus avoiding background binding of the positively charged fluorophore to dsDNA.

### Preparation of antibodies

α*-WH1*: Purified RepA-WH1(A31V)^18^ (200 μg) was mixed (1:1) with Freund’s complete adjuvant and intradermally injected into a New Zealand white rabbit. Three inocula were boosted at two week intervals. Pre- and post-immunization blood was collected and allowed to clot. Serum was collected by centrifugation and stored at −70 °C. IgGs (500 μl) were purified through a Hi-Trap rProtein A FF (1 ml) column run in a ÄKTA Basic-10 FPLC (GE Healthcare), pre-equilibrated in 20 mM Na-phosphate (pH 7.0). After washing the column with 4 volumes, IgGs were eluted in one step with 0.1 M Na-citrate (pH 3.0) and immediately neutralized adding 60 μl of 1 M Tris-HCl (pH 8.5). Animal handling was carried out at the animal facility of CIB-CSIC, upon approval by the Animal Welfare Committee of the Research Institute, according to the European and Spanish legal regulations.

*B3h7*: 500 μg of assembled RepA-WH1(A31V) amyloid fibres, sheared after several passages through the thin tip of a micro-pipette, were inoculated in four Balb/C mice (4 injections across 10 weeks). Antibody screening was initially performed by ELISA (SBA Clonotyping System/HRP, Southern Biotech) and confirmed by dot-blot (see below), leading to selection of the mouse exhibiting the highest antibody titres against the mixture of amyloid oligomers and fibres. Spleen cells from this mouse were fused with myeloma cells to form hybridomas^55^, which were cultured in HAT medium supplemented with Pen/Strep antibiotics (1,000 U ml^−1^; Life Technologies). The three hybridomas exhibiting the highest titres in ELISA/dot-blot went through two further subcloning rounds. The clone finally selected (848B3h7, in shorthand B3h7) was expanded in RPMI-1640 medium supplemented with 10% FCS and 2 mM Gln, and then used to produce ascitic fluid in five mice. The B3h7 antibody was purified from the ascites as described above for α-WH1. B3h7 isotype was identified as IgG2b by means of the Mouse Ig Isotyping Instant ELISA kit (eBioscience, Affimetrix). Antibody production, up to the stages of clonal amplification of hybridomas and obtaining of the ascites, was contracted to Abyntek Biopharma (Bilbao, Spain).

Purified IgGs were quantitated, after non-reducing SDS-PAGE and Coomassie blue staining, by densitometry of the bands using the Quantity One software (v. 4.6.3; Bio-Rad).

### Dot-blot assays

Nitrocellulose membranes (0.45 μm ø pore; Bio-Rad) were set in a Bio-Dot microfiltration device (Bio-Rad). Wells were pre-equilibrated with 0.1 mM Na_2_SO_4_, 40 mM HEPES (pH 8.0), 5 mM MgSO_4_, whereas those to be loaded with denatured proteins were rinsed with buffer supplemented with 1% methanol. Protein samples (0.2 μg, and subsequent 2-fold step dilutions) were either RepA-WH1(A31V) (a mixture of assembled oligomers and fibres), insulin fibres (2 mg ml^−1^, pH 1.2, heated at 60 °C for 2 h), microcine E492 fibres^56^, or purified RepA-WH1(A31V)-mCherry(mRFP) aggregates^21^. Samples were diluted in buffer (100 μl) and then spotted under gravity flow. RepA-WH2^12^ and yeast Orc4p-WH^33^,^34^ were also used as negative controls. For denatured samples, 0.1% of SDS was added to the dilution buffer and boiled for 4 min before blotting. Blotted membranes were then blocked, at room temperature for 1 h, with 2% BSA in Tris-buffered saline buffer (pH 7.0) containing 0.01% Tween-20 (TBS-T) and probed for another hour with the primary antibodies (1:1000 to 1:3000 in TBS-T), either in the Bio-Dot device or, if the membrane was removed, overnight at 4 °C. Besides α-WH1 and B3h7, the rabbit polyclonal antibodies A11 (anti-amyloidogenic oligomers; Invitrogen) or LOC (anti-amyloid fibres; Millipore) were used where indicated. The membranes were washed three times with TBS-T and then probed with the appropriate HRP-conjugated secondary antibodies (anti-mouse/rabbit; 1:10,000) for 1 h. After three additional washes with TBS-T, chemiluminiscent detection was performed on X-ray films with the ECL Prime kit (GE Healthcare). Western blot assays after SDS-PAGE were performed as described^23^,^34^.

### Fluorescence spectroscopy

Protein amyloidogenesis was also followed by means of thioflavin-T (Th-T) binding assays, by periodically removing from the incubation mixtures 25 μl aliquots, which were incubated with 60 μM Th-T (Sigma) in 475 μl of 0.1 M Na_2_SO_4_, 4 mM MgSO_4_, 20 mM HEPES (pH 8.0), at 25 °C for 15 min. Fluorescence emission spectra was monitored with an excitation wavelength of 440 nm (excitation slit 5 nm, emission slit 10 nm) using a Fluoromax-2 spectrofluorometer (Jobin Yvon-Spex, Horiba). The spectrum for the buffer was then subtracted as a baseline.

### Peptide arrays

Dodecapeptides spanning the whole sequence of RepA-WH1, with an overlap of ten residues (Fig. 2c), were solid-phase synthesized (Fmoc) and immobilized (≈20 nmol per spot) in an Amino-PEG_500_-UC540 sheet at the Proteomics facility of the National Centre for Biotechnology (CNB-CSIC, Madrid), as described^57^. To enhance solvation of hydrophobic peptides, the membranes were briefly rinsed with ethanol. Membranes were washed three times with TBS and incubated with 5% blocking solution (ECL Advance blocking agent; GE Healthcare) in TBS-T for 4 h. Membranes were then probed overnight with primary antibodies (B3H7 or α-WH1, 4.4 ng) and washed three times with TBS-T. The secondary antibodies (HRP-conjugated anti-mouse/rabbit; 1:10,000) were incubated for 2 h, and then blots were washed three times with TBS-T and developed with the ECL Advance reagent (GE Healthcare). For membrane stripping, blots were sequentially incubated with 8 M urea, 1% SDS, 0.5% β-mercaptoethanol in PBS for 30 min at 55 °C and three times with acetic acid/ethanol/Milli-Q water (10:50:40). Membranes were then washed with TBS and incubated with blocking solution before re-hybridization. For each spot, both the antibody signal and peptide load (Ponceau red staining) were quantitated as indicated above for the purified antibodies.

### Electron microscopy

*iEM of amyloid fibres*: Samples adsorbed on carbon-coated and glow-discharged 400-mesh copper grids (Ted Pella) were successively floated on drops containing blocking solution (BS: 2% BSA, 0.05% Tween-20 in PBS) for 30 min and the mouse B3h7 or rabbit α-WH1 primary antibodies (0.4 ng μl^−1^ in BS, for 1 h at room temperature). After three washes (10 min each) with 0.05% Tween-20 in PBS, grids were incubated with gold-conjugated (10 nm ø particles) anti-mouse/rabbit secondary antibodies (Sigma; 1:50 in BS), and then washed three times with 0.05% Tween-20 in PBS and once with bi-distilled water. Grids were then air-dried and contrasted with 2% uranyl acetate for 2 min. Unlabelled RepA-WH1(A31V), insulin and microcin E492 amyloid fibres were processed as described^18^.

*iEM of bacterial cells*: Cultures of *E. coli* MC4100 expressing the prionoid RepA-WH1(A31V)-mCherry(mRFP)^23^ were harvested 2–4 h after dilution of exponential inocula. In parallel, control samples were taken from cultures not expressing the prionoid. Cells were fixed, embedded in resin, sliced, sequentially incubated on the grids with the primary antibodies (0.2 ng μl^−1^) and the secondary Au-conjugated antibodies and stained with uranyl acetate, as described^21^,^23^. Specimens were then examined in a JEOL JEM-1230 transmission electron microscope, operating at 100 kV, and images were captured with a TVIPS TemCam-F416 CMOS camera.

## Additional Information

**How to cite this article**: Moreno-del Álamo, M. *et al.* Pre-amyloid oligomers of the proteotoxic RepA-WH1 prionoid assemble at the bacterial nucleoid. *Sci. Rep.* **5**, 14669; doi: 10.1038/srep14669 (2015).
